# Human cytomegalovirus inhibits the proliferation and invasion of extravillous cytotrophoblasts via Hippo-YAP pathway

**DOI:** 10.1186/s12985-021-01681-2

**Published:** 2021-10-30

**Authors:** Qiaoqiao Kong, Jing Li, Li Zhao, Peng Shi, Xiaobei Liu, Cailing Bian, Jing Liu, Tao Liu

**Affiliations:** 1 Post-Doctoral Working Station, Tai’an City Central Hospital, Tai’an, Shandong China; 2 Department of Reproduction and Genetics, Tai’an City Central Hospital, Tai’an, Shandong China

**Keywords:** Human cytomegalovirus, Extravillous cytotrophoblasts, Hippo-YAP pathway, YAP

## Abstract

**Background:**

Human cytomegalovirus (HCMV) infection in utero is very common during pregnancy, which can lead to adverse outcomes in both pregnancy and progeny, but its pathogenesis has not been fully clarified. The decrease of extravillous cytotrophoblasts (EVT) invasion is an essential pathophysiological process of some pregnancy complications. Hippo-YAP signaling pathway plays an important role in regulating cell proliferation and apoptosis. However, whether YAP is involved in HCMV uterine infection remains to be studied.

**Methods:**

The primary EVT was cultured and infected by the HCMV strain AD169 virus in vitro. Immunofluorescence staining of HCMVpp65 antigen was conducted afterward to confirm the establishment of an infection model. The optimal virus infection dose was determined by the EVT proliferation status in vitro. Real-time PCR was performed to examine the mRNA level of major genes involved in the Hippo pathway in EVT after HCMV infection. The effect of HCMV on the expression of YAP protein in EVT was evaluated by Immunofluorescence staining and Western blot. An in vitro cell invasion assay was carried out to analyze the influence of HCMV on EVT invasion. The changes of EVT invasion was accessed by establishing YAP silencing and over-expression models using YAP1 specific siRNA and plasmid pcDH.

**Results:**

The optimal HCMV infection dose was 282.5TCID50/ml. Compared to the control group, the infection of HCMV significantly reduced the mRNA expression of Mst1, Mst2, SAV, Lats1, Lats2, Mob1, YAP1, TAZ, TEAD1-4 genes and YAP protein expression in the Hippo-YAP pathway. HCMV infection also decreased the EVT invasion. In non-infected EVT, the number of transmembrane EVT cells was significantly reduced when YAP1 gene was silenced, while it was significantly increased when YAP1 gene was over-expressed. In the HCMV-infected EVT, the number of transmembrane EVT cells significantly increased when over-expressed and eventually recovered to the level of NC.

**Conclusions:**

HCMV may decrease EVT invasion by inhibiting the expression of mRNA and protein of YAP in the Hippo-YAP signaling pathway. HCMV eventually reduces the invasion ability of EVT by inhibiting multiple genes in the Hippo-YAP signaling pathway, especially inhibiting YAP which serves as the downstream effector.

## Introduction

Human cytomegalovirus (HCMV), also known as human herpesvirus 5 (HHV-5), belongs to the Herpesviridae family, subfamily Betaherpesviridae. It is the virus with the largest nucleic acid molecular weight in the Betaherpesvirinae subfamily. HCMV commonly exists in nature and has a high level of host specificity. Humans are its only host [1]. In the United States and Europe, the infection rate of human cytomegalovirus is 40–60%. 1–4% of women in childbearing age have primary HCMV infection during pregnancy. Among them, 30% to 40% of infection cases could develop into intrauterine infection [2]. At present, HCMV is considered as the most common pathogen causing active infections, intrauterine infections of embryos or fetuses in pregnant women. Intrauterine transmission after HCMV infection in pregnant women can lead to congenital infections of embryos and fetuses, causing abnormal pregnancy outcomes such as embryo suspension, abortion, retardation of fetal growth, microcephaly and stillbirth. Also, neonatal sensorineural deafness, cognitive under-development and other serious consequences can be seen in infected newborn babies [3].

In a normal pregnancy, in order to maintain adequate blood flow to the embryo or fetus, extravillous cytotrophoblast (EVT) remodels uterine spiral arteries through proliferation, migration and invasion of uterine decidua as well as superficial muscle layer. The decrease in proliferation of EVT will reduce its invasion ability, which negatively affects the remodeling of uterine spiral arteries. The poor remodeling of spiral arteries causes insufficient blood supply to the placenta, resulting in abnormal pregnancy outcomes such as miscarriage, stillbirth, intrauterine growth retardation, and gestational hypertension [4]. Current study suggested that that HCMV infection of trophoblast cells is the initial step of intrauterine infection [5, 6].

The Hippo signaling pathway was first identified in Drosophila. It is also a highly conserved signaling pathway; Recent studies have proved that the Hippo pathway is widely present in mammals. Under normal physiological conditions, YAP (Yes-associated protein, YAP) is expressed in all human tissues and cells except leukocytes. It is especially common in the placenta, prostate, ovarian tissues. Hippo signaling pathway can regulate the size of organs and tumor formation. During normal cell growth, YAP/TAZ binds to the transcription factor TEAD family in the nucleus, initiating the expression of downstream target genes, therefore to promote the cell growth or inhibit the cell apoptosis. Over-expression of YAP protein can cause excessive cell proliferation resulting in abnormal organ enlargement and tumor occurrence; Vice versa, inactivation of YAP may inhibit proliferation of cells, leading to atrophy of tissues and organs [7]. In fact, trophoblast cells play the role of a tumor-like cells in the process of embryo implantation and placenta formation [8]. However, whether the Hippo-YAP signaling pathway is involved in HCMV-induced downregulation of invasion and proliferation of EVT remains unknown.

HCMV infection in pregnant women can lead to miscarriage, stillbirth, and intrauterine growth retardation. However, only a limited number of studies have proved that HCMV decreases the EVT proliferation and invasion ability [9–12], which may have a further adverse effect on pregnancy outcomes. Yet, the exact molecular mechanism is still unclear. Therefore, the purpose of this study is to assess the role of the Hippo-YAP signaling pathway on HCMV induced low EVT proliferation and invasion by establishing an in vitro model of HCMV intrauterine infection. Further to clarify the underlying molecular mechanism of HCMV intrauterine infection.

## Materials and methods

### Tissue samples and virus

Placenta samples were collected from healthy pregnant women (peripheral HCMV IgM negative) who voluntarily terminate the pregnancies in the first trimester (5–10 weeks). Samples were obtained from the Department of Obstetrics and Gynecology in Tai’an City Central Hospital between July 2016 and July 2018. All experimental procedures were approved by the medical ethics committee of the Tai’an City Central Hospital and patients informed consent agreement. HCMV AD169 was provided by the Hubei Provincial Institute of Virology and the toxicity was 10^5.15^ TCID50/ml.

### Isolation and culture of primary EVT

Isolation and primary culture of EVT was performed according to the protocol of Liu et al. [11]. The human chorionic villi were washed with D-Hank’s to remove the decidua and matrix. The tissue was then cut into 1 mm^3^ pieces and incubated in enzyme mixture containing 4.2 mM MgSO4, 0.125% trypsin, 20 U/ml DNase I and 25 mM Hepes at 37 °C for 50 min. The 80 mesh (177 micron) and 300 metal mesh was used to filter the supernatant. The filtrate was centrifuged at 500 × g for 10 min and the pellets were suspended in DMEM/F12 medium. 35%, 40%, 45% or 50% Percoll gradient solution were supplemented into the cell suspension and centrifuged at 1000×*g* for 25 min. The suspension from 40 to 45% Percoll gradient was washed twice and suspended in DMEM/F12 medium containing 10% FBS. The cell suspension was inoculated in 48 well-culture plates at a density of 5 × 10^4^cells/cm^2^and cultured in 37 °C, 5% CO_2_ for 24 h. The cells were then washed 3 times with sterile D-Hank’s and fresh DMEM/F12 medium was added. More than three independent isolated cell culture were conducted and the purity of isolated EVTs were analyzed in each isolation. In our study, there was no significant difference in the proliferation properties of different isolation.

### EVT infection by HCMV

EVTs were inoculated in 6-well plates at a density of 5 × 10^4^/cm^2^ and cultured at 37 °C, 5% CO_2_ for 24 h. Then the DMEM/F12 medium was replaced with the HCMV culture medium. The virus group was inculated with 4 μL HCMV (TCID50:10^–4.15^/0.1 mL) for 2 h and the normal group was treated with PBS. The HCMV medium was discarded and cells were washed for 3 times with sterile D-Hank’s. The EVTs were cultured in fresh culture medium for 24 h or 48 h at 37 °C, 5% CO_2_. Immunofluorescence staining was used to detect HCMVpp65 antigen.

### MTT assay

Cells were inoculated in 96-well plates at a density of 10^3^/ml. 200 μL of cell solution was added in each well and a blank control was added for each plate. The EVT culture medium was replaced with120 μL virus culture medium for each well. For the virus group, 10, 20, 30, 40, 50, 70 and 100 mol/L HCMV solutions were added to each well and the control group received the same volume of PBS. Two hours later, virus culture medium was replaced with 150 μL EVT culture medium.20 μL MTT solution was added to each well at 24 and 48 h respectively, and cells were cultured for another 4 h. The medium was discarded and 150 μL DMSO were added to each well. The plate was shaken for 10 min. The OD value at 490 nm was measured to analyze the effects of HCMV on EVT proliferation. The minimum viral titer with a significant effect on cell proliferation was considered to be the optimal viral titer for inoculation.

### Quantitative real-time polymerase chain reaction (qRT-PCR)

Total RNA of EVT from two groups was extracted with an RNA extraction kit (Takara, Japan). RNA was transcribed into cDNA using a reverse transcription kit (Takara, Japan). According to the sequences of genes, qRT-PCR primers were designed (Table 1). β-actin was designed as the internal standard. Quantification of mRNA was conducted using the Mx3005P real-time PCR instrument (Stratagene, Valencia, CA). The PCR reaction system contained 10 μL 2*SYBR Green general qPCR Master Mix (TaKaRa, Japan), 8.8 μL 1:100 diluted cDNA and 0.6 μL upstream/downstream primer, respectively (10 μM). Cycle amplification conditions comprised an initial denaturation step at 95 °C for 30 s followed by 40 cycles at 95 °C for 3 s and at 60 °C for 30 s. Gene expression was normalized to β-actin internal control. All values were then expressed relative to control samples using the 2^–(ΔΔCT)^ method.

**Table 1:** Primer sequences information

Name of primers	Primers sequences (5′–3′)
Mst1	
Forward	CCCTGGGAATAACTGCCATA
Reverse	ATGAAGATTGCCCTCATTGG
Mst2	
Forward	GCTTGGAGAAGGGTCTTATGG
Reverse	CATATGGGCTGTCACATTGC
Sav	
Forward	CACACAAATAAGAAGGCCCAA
Reverse	TGGCTGGTATGTGACAGGAG
Lats1	
Forward	ATACTTGGGGTTGCTGGGAC
Reverse	ATTAACTCTGGAGGGGAGAGCA
Lats2	
Forward	CTCCGCAAAGGGTACACTCA
Reverse	GAGCGTGTTCTCCCAGTTGA
Mob1	
Forward	TTCCAGAGGGTTCTCACCAG
Reverse	CACAGTGTTAACTGCAACCCA
YAP	
Forward	GCAGTTGGGAGCTGTTTCTC
Reverse	GCCATGTTGTTGTCTGATCG
TAZ	
Forward	CAGCCAAATCTCGTGATGAA
Reverse	TTCTGCTGGCTCAGGGTACT
TAED1	
Forward	GCCCTGGCTATCTATCCACC
Reverse	TAGACACCTGTTTTCTGGTCCTC
TAED2	
Forward	AGGCTTTCCAGACAATGGCA
Reverse	AAAAGCTCAGAGGCCTGGAC
TAED3	
Forward	GACCCTGACACGTACAGCAA
Reverse	GAGCTCCTTCAATCCTCCCT
TAED4	
Forward	GAGCAGAGTTTCCAGGAGG
Reverse	TCGTTCCGACCATACATCTT
β-actin	
Forward	CATCCGTAAAGACCTCTATGCCAAC
Reverse	ATGGAGCCACCGATCCACA

### Immunocytochemistry (fluorescence) staining

Plastic coverslips placed in 6 well-culture plates was coated with mouse tail glue and primary EVTs were inoculated in above 6-well plates for 48 h. Then coverslips were collected, washed three times with PBS and fixed for 30 min in a methanol-acetone (1:1) solution at room temperature. The coverslips were treated with 3% H_2_O_2_ for 20 min, 0.3% Triton X-100 for 30 min and 10% BSA for 30 min. For immunocytochemistry staining, EVTs were incubated in mouse anti-human CK7, anti-YAP, anti-Vim monoclonal antibody, rabbit anti-c-erbB-2 polyclonal antibody (1:100 diluted, Santa Cruz Biotechnology, China) at 4 °C overnight. ALP-conjugated goat-anti-mouse IgG or goat-anti-rabbit IgG (1:50 diluted, Santa Cruz Biotechnology, China) at 37 °C for 1 h. EVTs were stained with DAB (3,3′-diaminobenzidine) straining kit (Sangon Biotech, China) and the nucleus was strained with hematoxylin. For immunofluorescence staining, EVTs were incubated in rabbit anti-HCMVpp65 polyclonal antibody (1:100 diluted, Santa Cruz Biotechnology, China) at 4 °C overnight, and then, incubated at 37 °C for 1 h in Cy3-conjugated goat-anti-rabbit IgG (1:50 diluted, Santa Cruz Biotechnology, China) and incubated for 10 min with 10 µg/ml Hoechst 33,342. Finally, coverslips were washed twice with PBS and flipped (the cell side now facing down) on top of 25 mL droplot of PVA-DABCO® mounting solution (Sigma-Aldrich) on glass slides. Straining observed with a microscope. Staining of EVTs were observed with a Leica laser scanning confocal microscope or the inverted microscope. The positive signal of antibody in immunocytochemistry staining appears as brown particle Fluorescence was detected with bandpass emission filters: 420–480 nm for Hoechst and 560–605 nm for Cy3, and the captured signals were recorded as blue, green and red, respectively. To quantify YAP expression in EVTs, the relative fluorescence intensities were measured on the raw images using Image-pro Plus software (Media Cybernetics Inc, Silver Spring, MD) under fixed thresholds across all slides.

### Western blot

The total protein was extracted and the concentration was measured using a total protein extraction kit (BestBio, China), according to the manufacturer’s instructions. 50 μg of total protein from each sample was separated on a 10% SDS-PAGE gel by electrophoresis and transferred to a PVDF membrane. The membrane was blocked in 5% skim milk at room temperature for 2 h and then incubated with mouse anti-YAP monoclonal antibody (1:500 dilution) overnight at 4 °C. The membrane was then washed in TBST and incubated with the secondary antibody at room temperature for 2 h. The membrane was washed again in TBST, incubated with ECL reagent and protein bands were visualized under a gel imager. Gel image Quantity One analysis software was used to measure the absorbance values of the YAP protein and the internal control β-actin protein. The mean absorbance of the target protein over that of the internal control was used as the relative expression level of the target protein.

### In vitro invasion assay

In vitro invasion assay was used for determining the invasive potential of EVTs. The Transwell chamber coated with Matrigel was placed in 24-well culture plates and 400 μL medium containing conditioned medium and complete medium (1:1) was infused into well out of chamber. In the HCMV group, EVT at 1 × 10^5^/mL and 100 TCID50 HCMV 14.29 μL (with a total amount of 100 μL) was used in the Transwell chamber. In the control group, an identical volume of PBS was used instead of HCMV solution. After incubation for 24 h, the sample was fixed with formaldehyde, hematoxylin-stained and observed under an inverted microscope to count the number of cells migrating through the micropore membrane. For each sample, 10 randomly selected high power fields were counted.

### siRNA and plasmid transfection

The YAP specific siRNAs and pcDH plasmid including negative control were designed and synthesized by RiboBio (Guangzhou, China). When cells grew to 50%–70% of confluence, transfection was performed according to instructions of X-tremeGENE™ HP DNA tansfection reagent and X-tremeGENE™ siRNA tansfection reagent (Roche, Switzerland). For plasmid transfection, 1 μg or 0.5 μg pcDH was mixed with 200 μl opti-MEM in a sterile tube. Add 3 μl or 1.5 μl X-tremeGENE HP DNA Transfection Reagent to the diluted DNA respectively (3:1 ratio of reagent to DNA) and mixed. For siRNA transfection, 0.66 μg or 1 μg siRNA was mixed with 50 μl opti-MEM in a sterile tube. Add 2 μl or 3 μl X-tremeGENE HP siRNA Transfection Reagent to the diluted siRNA respectively (3:1 ratio of reagent to DNA) and mixed. The above mixture was incubated for 15–30 min at room temperature. 200 μl transfection complex was added to the cells in a dropwise manner and cells were incubated for 24 h before measuring YAP1 mRNA level.

### Statistical analysis

In all the experiments, each treatment was repeated at least three times. Data were analyzed using ANOVA when each measure contained 3 or more groups or using Independent-Samples T Test when each measure contained only two group. For ANOVA, a Duncan multiple comparison test was used to determine differences. The SPSS 18.0 software package was used for statistical analysis and data were expressed as mean ± SEM. *P* < 0.05 was considered to be statistically significant.

## Results

### Assay of HCMV AD169 toxicity

According to the data in Table 2, The TCID50 of HCMV AD169 strain is 10^–4.15^/0.1 ml calculated by Reed-Muench method, that means the toxicity was 10^5.15^ TCID50/ml.

**Table 2:** Assay of HCMV AD169 toxicity by virus titration method

Dilution rate of HCMV	Cytopathic ratio	Cytopathic rate (%)
10^–1^	22/22	100.0
10^–2^	16/16	100.0
10^–3^	10/10	100.0
10^–4^	4/7	57.1
10^–5^	1/9	11.1
10^–6^	0/14	0.0
10^–7^	0/20	0.0
10^–8^	0/26	0.0

### The purity of isolated EVTs

**Fig. 1:**
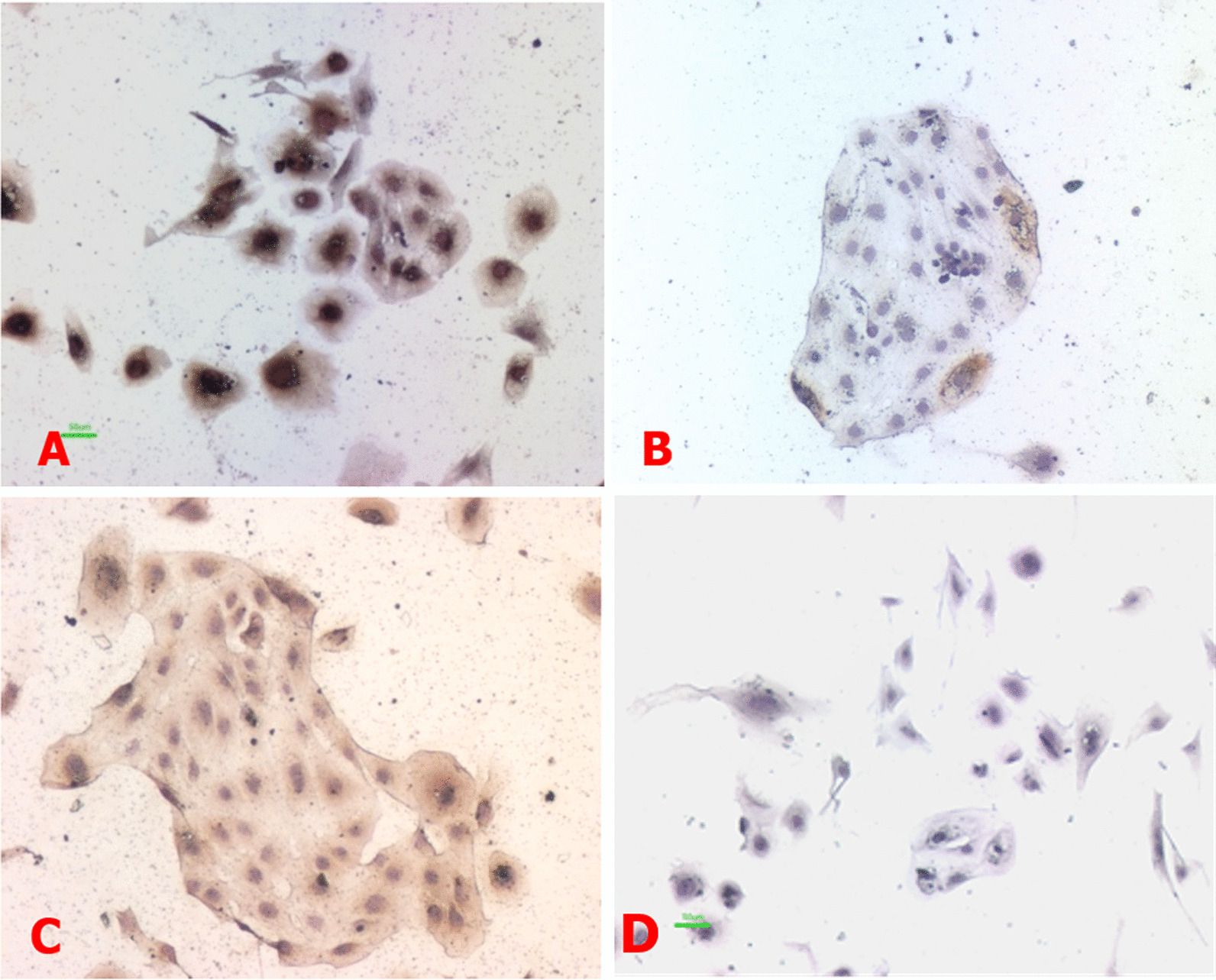
Immunohistochemical staining of CK7, Vim and c-erbB-2 antigens in EVT. **A–D**. CK7, Vim, c-erbB-2 antigens and negative control were detected, respectively × 100. The experiments were repeated at least 3 times

Immunocytochemistry staining showed that isolated primary EVTs were mononuclear, triangular or irregular in shape and almost all of the cells were positively stained for CK7 (Fig. 1A) and c-erbB-2 (Fig. 1C), indicating the high purity of primary EVT isolated from human chorionic villi. The occasional stain of Vim could be from fibroblasts (Fig. 1B).

### In vitro HCMV infection of EVT

**Fig. 2:**
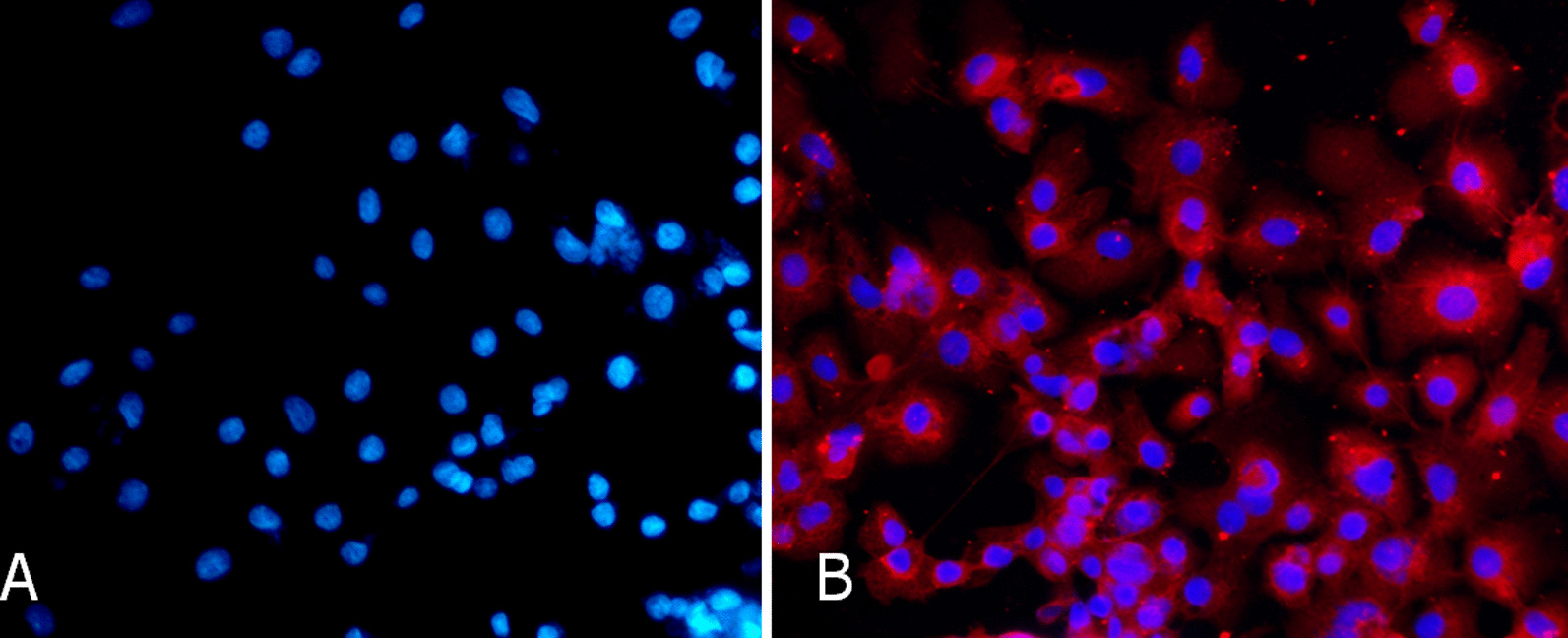
Immunofluorescence detection of HCMV pp65 antigens in EVT. **A** Control group (Nucleus stained blue; no red signal); **B** Virus group (HCMV pp65 antigen stained red; nucleus stained blue) × 200. The experiments were repeated at least 3 times

Immunofluorescence staining showed that a large number of red HCMVpp65 antigen signals were expressed in the cytoplasm of the HCMV-infected EVT (Fig. 2A); while it was not seen in the control group (Fig. 2A). The nuclear staining was in blue in both groups. These results suggested that HCMV could infect EVT in vitro and replicates in EVT cells. The establishment of an in vitro model of HCMV-infected EVT was successful.

### Effect of HCMV on EVT proliferation

**Fig. 3:**
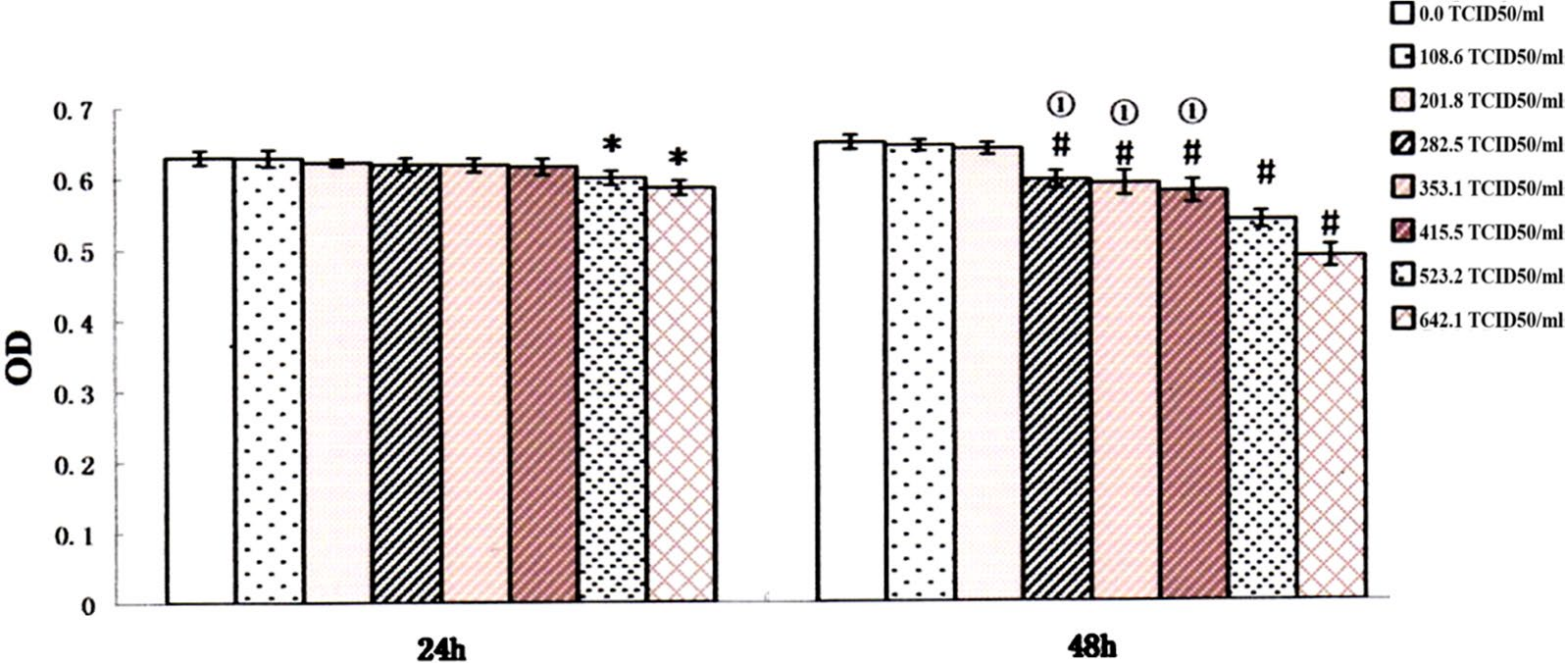
EVT proliferation following culture in HCMV solution with different concentrations for 24 h or 48 h. * and # indicates significant (*P* < 0.05) difference from control values and ① indicates no significant (*P* > 0.05) difference between the three groups. The experiments were repeated at least 3 times

Infection with 108.6 to 415.5 TCID50/ml virus for 24 h and with 108.6 or 201.8 TCID50/ml virus for 48 h had little effect on the proliferation of EVT (*P* > 0.05). However, EVT proliferation was significantly inhibited when cultured in 523.2 or 642.1 TCID50/ml virus solution for 24 h or in 282.5 to 642.1 TCID50/ml virus solution for 48 h (*P* < 0.05). No significant differences were observed in the EVT proliferation after infection with 282.5, 353.1 or 415.5 TCID50/ml HCMV for 48 h. (Fig. 3). Based on the above result, 282.5 TCID50/ml HCMV was selected for subsequent experiments.

### Effect of HCMV on EVT invasion

**Fig. 4:**
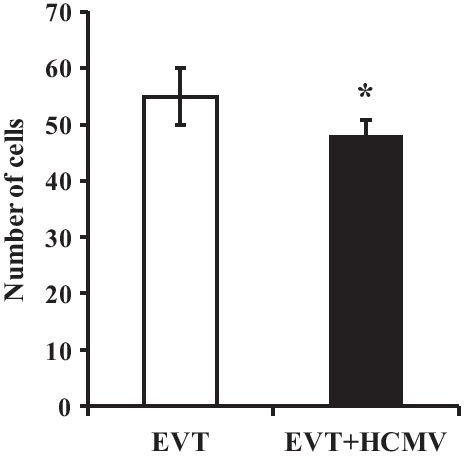
The effect of HCMV infection on EVT invasion. * indicates significant (*P* < 0.05) difference from control values. The experiments were repeated at least 3 times

EVT invaded the Matrigel in both the HCMV-infected group and the control group. However, the number of EVT penetrating Matrigel in the HCMV-infected group was significantly lower (47.9 ± 3.21) than that in the control group (55.7 ± 2.95), suggesting that HCMV reduced EVT invasion (*P* *<* 0.05) (Fig. 4).

### Effect of HCMV on the expression of major genes in Hippo-YAP signaling pathways in EVT

**Fig. 5:**
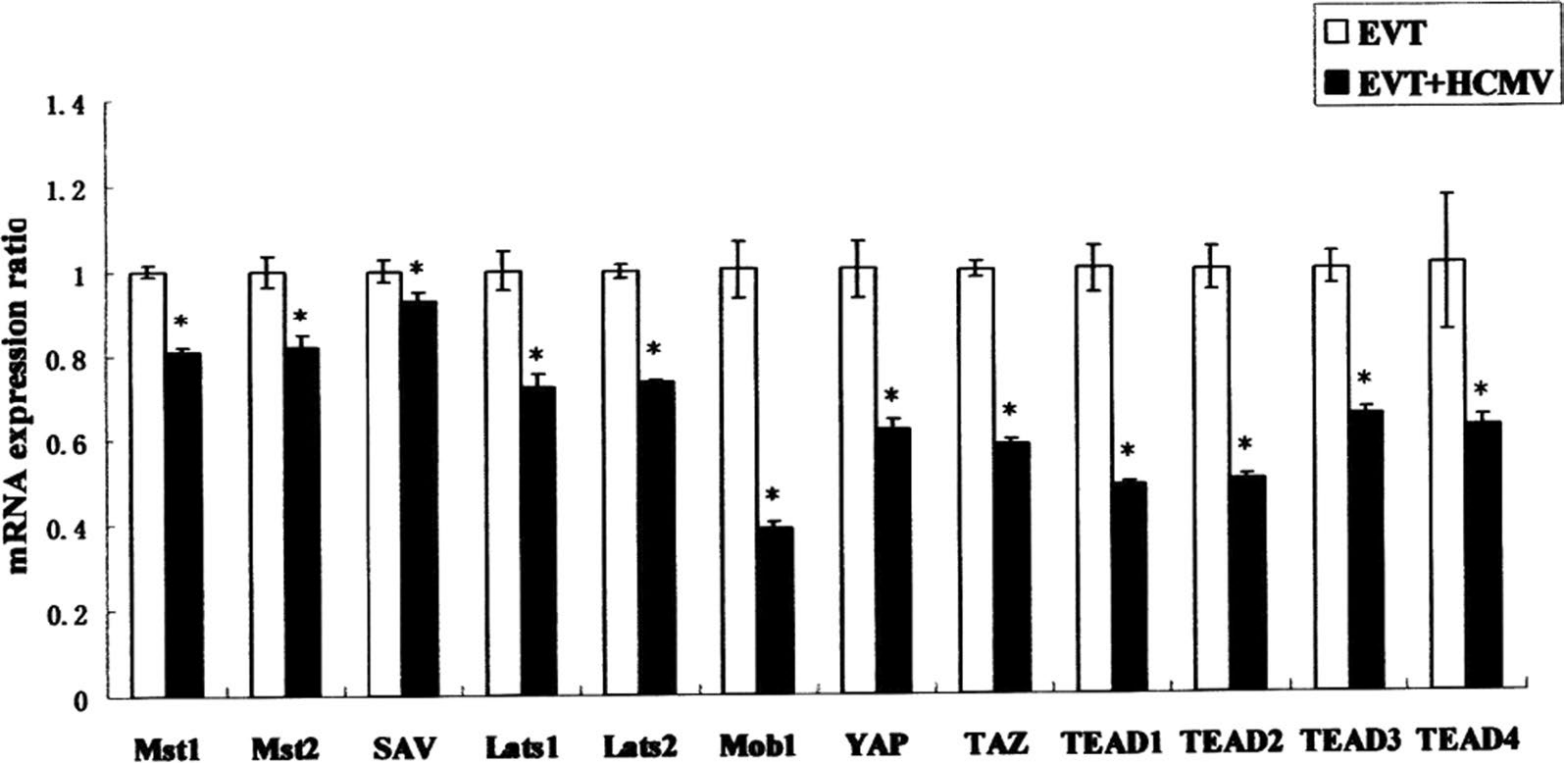
Effect of HCMV on mRNA expression of major genes in Hippo-YAP signaling pathways in EVT to be detected by qRT-PCR. * indicates significant (*P* < 0.05) difference from control values. The experiments were repeated at least 3 times

The qRT-PCR shows that the expression of Mst1/2, SAV, Lats1/2, Mob1, YAP, TAZ, TEAD1 ~ 4 mRNA were all decreased in HCMV-infected EVT compared to the control group (*P* < 0.05) (Fig. 5).

### Effect of HCMV on the expression of YAP protein in EVT

**Fig. 6:**
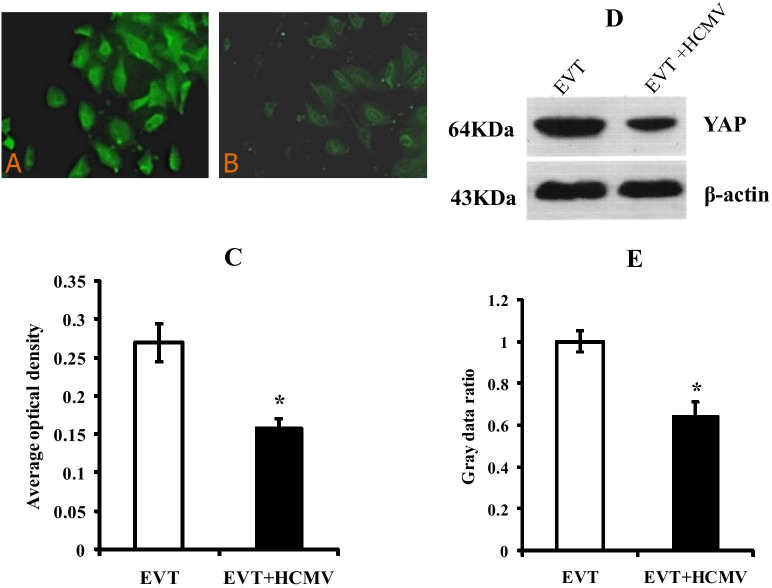
Effect of HCMV on YAP protein expression in EVT. **A** and **B** showed YAP immunofluorescent staining in the control EVT (200×) and HCMV-infected EVT (200×), respectively; **C** is comparison of fluorescence intensity of YAP between the control and virus groups; **D** shows YAP Western blots in the control and virus groups; **E** shows levels of YAP by Western blot quantification in EVT from the control and virus groups. * indicates significant (*P* < 0.05) difference from control values. The experiments were repeated at least 3 times

Immunofluorescence staining showed green signal of YAP protein in both the control group (EVT) and the HCMV-infected group (EVT + HCMV). The YAP protein expressed in the cytoplasm and nucleus. In the HCMV-infected group, the intensity of the YAP fluorescence signal was weaker than the control group and the nuclear location of the YAP protein was reduced (Fig. 6A, B). The results of the semi-quantitative analysis demonstrated that the expression of YAP protein in HCMV-infected EVT was significantly lower than the control group (*P* < 0.05) (Fig. 6C). Western blot showed that YAP protein was significantly decreased in infected EVT (*P* < 0.05) (Fig. 6D, E).

### Effect of knockdown or over-expression of YAP on EVT invasion

**Fig. 7:**
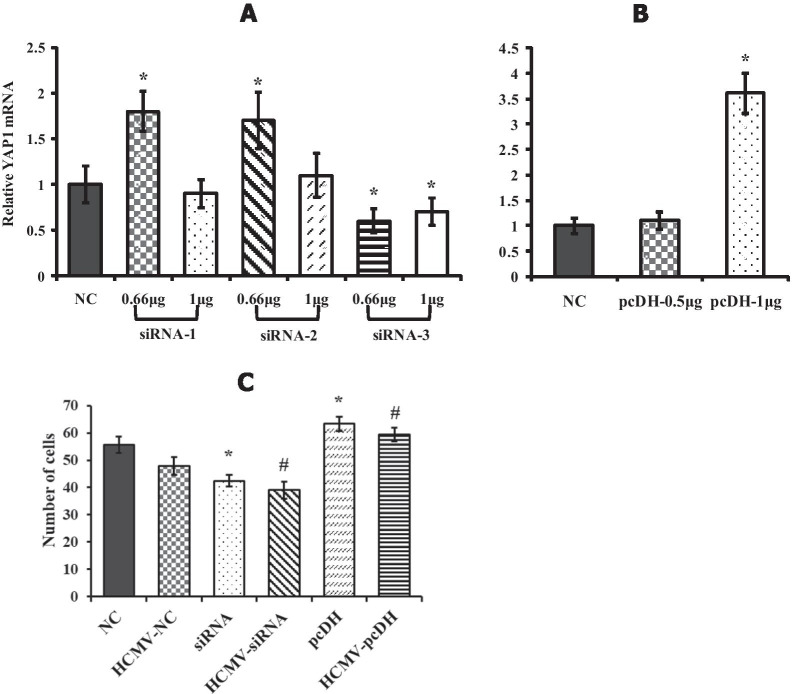
Effect of YAP-knockdown or YAP-overexpression on EVT invasion. **A** and **B** shows mRNA expression of YAP1 in EVT after transfection for 24 h with 3 different siRNA sequence and pcDH plasmid at different concentrations, respectively. **C** shows number of EVT cells which invaded the Matrigel after transfection with 0.66 μg siRNA-3 or 1 μg pcDH. NC is negative control group. * indicates significant (*P* < 0.05) difference from NC group and # indicates significant (*P* < 0.05) difference from HCMV-NC group. The experiments were repeated at least 3 times

In order to confirm that HCMV suppresses the invasion of EVT via inhibiting the expression of YAP, a cell model of YAP1-silencing and YAP1-overexpression was established through siRNA technology and lentiviral vector (pcDH), respectively, to determine its effect on invasion of EVT. Preliminary experiments showed that efficiency was the highest transfected in 0.66 μg siRNA-3 and 1 μg pcDH for 24 h. Therefore 0.66 μg siRNA-3 and 1 μg pcDH was used for the following transfection experiment. The cell invasion experiments showed that EVT penetrated Matrigel in all groups. The number of transmembrane cells in the non-infected group was significantly reduced after silencing of YAP1, but significantly elevated after over-expression (*P* < 0.05) (Fig. 7C, NC vs siRNA vs pcDH). In the HCMV-infected group, the number of transmembrane cells was significantly decreased after transfecting the EVT with YAP1-siRNA (Fig. 7C, HCMV-NC vs HCMV-siRNA). Whereas, after transfecting the EVT with pcDH, the invasion of EVT increased significantly (Fig. 7C, HCMV-NC vs HCMV-pcDH, *P* < 0.05) and eventually recovered to the level of the control group (Fig. 7C, HCMV-pcDH vs NC, *P* > 0.05). The above results proved that HCMV reduced the invasion ability of EVT via suppressing the expression of YAP.

## Discussion

Primary EVT culture provides an appropriate method to explore the mechanisms of HCMV on the proliferation and invasion of extravillous cytotrophoblasts. CK7 is a cytoskeletal protein of epithelial cells, c-erbB-2 is an epidermal growth factor receptor with tyrosine kinase activity and can be used as a specific EVT marker and Vim is a marker of endothelial and stromal cells but is not expressed in EVTs [13]. In our study, healthy early chorionic villi and isolated primary EVTs were collected. Immunocytochemistry staining showed that the isolated primary EVTs were mononuclear and majority of cells were triangular or irregular in shape. Almost all of the cells were positively stained for CK7 and c-erbB-2, and occasionally Vim positive. This suggests the high purity of primary EVT isolated from human chorionic villi, thus laying the foundation for future experiment.

HCMV is widely distributed, and humans are the only host. Several HCMV strains have been identified according to the specificity of antigen. The HCMVpp65 antigen is an early indicator of HCMV infection due to its peripheral blood lymphocytes, expression in vascular endothelial cells, polymorphonuclear leukocytes and monocytes within 6–24 h after infection [14, 15]. In this study, immunofluorescence staining showed that HCMVpp65 antigen expressed in the HCMV-infected primary EVT, but not in the control group, indicating HCMV could infect EVT in vitro and replicates in EVT cells.

HCMV intrauterine infection can cause serious consequences such as miscarriage, stillbirth, and fetal growth retardation [16]. At present, it remains unclear how HCMV intrauterine infection leads to abnormal development of embryos and fetuses. It is believed that HCMV infection of trophoblast cells is the initiating step of intrauterine infection [17, 18]. After implantation of blastocysts, villous trophoblast cells differentiate into villa trophoblasts and EVT. EVT proliferates and migrates to form a cell column, which aggressively grows into the uterine interstitium and spiral artery cavity to form fixed villi [19]. So far, HCMV infects different types of trophoblast cells, including CTs, STs, VTs and EVT, leading to cell damage and death, poor differentiation of CTs, impaired VT fusion, diminished numbers of and immune secretion dysfunction in STs, and reduced the proliferation and invasion of EVT [5, 9, 22–25]. The decrease of EVT proliferation will reduce its invasion ability. Notably, the decrease of EVT invasion is an essential pathophysiological process of miscarriage, stillbirth, intrauterine growth retardation, pregnancy-induced hypertension, and other diseases [20, 21]. In our study, proliferation and invasion of EVT were significantly decreased after infected with HCMV compared with normal EVT. The result suggested that HCMV may cause adverse pregnancy outcomes through impairing proliferation and the invasion of EVT.

The Hippo signaling pathway has an essential role in regulating cell proliferation and organ size and development. YAP plays a key role in the signaling pathway as a transcriptional co-activator. In mammals, this pathway consists of Mst1/2 kinase, Sav, Lats1/2 kinase, Mob1 and YAP. The pathway regulates as follow: Mst1/2 binds to Lats1/2 and Mobl complexes through Sav, which activates Lats1/2 and Mobl complex. The activated Lats1/2 then phosphorylates the 127th serine site of YAP (YAPS127) and therefore to regulates YAP activity. YAP binds to the transcription factor TEAD in the nucleus activating the expression of downstream target genes. This promotes the cell proliferation and inhibits cell apoptosis, therefore to regulate organ size and tumor formation [26]. In fact, the proliferation and invasion of EVT is essential for the normal fetal development [20, 21] and trophoblast cells participate as tumor-like cells in the process of embryo implantation and placenta formation [8]. With these in mind, whether the Hippo-YAP signaling pathway involves in the damage of HCMV to the proliferation and invasion of EVT cells is unclear. In our study, the mRNA level of Hippo-YAP signaling pathway-related genes, including Mst1/2, Sav, Lats1/2, Mob1, YAP, TAZ, TEAD1-4, were significantly reduced in the HCMV-infected group compared with the control group. Immunofluorescence staining and Western blot showed that EVT cells express YAP protein and the expression level of YAP protein in HCMV-infected EVT was significantly lower than that in the control group. In summary, the decrease of EVT cell proliferation and invasion in HCMV-infected EVT accompanied by the inhibition of the Hippo-YAP signaling pathway. According to the above results, we could speculate that HCMV may influence the invasion and proliferation of EVT through the Hippo-YAP signaling pathway, in which YAP plays a key role as a downstream effector.

In order to further affirm that HCMV suppresses the invasion of trophoblast cells via affecting the expression of YAP, we further detected invasion function of EVT after YAP1 silencing and overexpression. The results of in vitro cell invasion experiments showed that the invasion of EVT in the non-infected group was significantly reduced after YAP1 gene silencing, but significantly elevated after overexpression. In the HCMV-infected group, the invasion of EVT was significantly decreased after transfecting EVT with YAP1-siRNA, but increased when overexpressed. The above results prove that HCMV reduces the invasion ability of EVT via suppressing the expression of YAP.

YAP has a nuclear translocation mechanism in cells. In the Hippo signaling pathway, LATS1/2 induces phosphorylation of MST1/2, which in turn induces thephosphorylation of YAP. The phosphorylated YAP can bind to14-3–3 protein in the cytoplasm and stays in the cytoplasm, which eventually leads to YAP degradation or ubiquitination, thereby losing its activity [27]. The unphosphorylated YAP accumulates in the nucleus and interacts with DNA-binding transcription factor TEA to induce transcription program [26]. YAP/TAZ promotes cell growth or inhibits cell suppression by binding to transcription factor TEAD which located in the nucleus and activating the expression of downstream target genes. This process can regulate the size of organs and tumor formation [28]. In this study, the nuclear translocation of YAP was also observed. The intensity of the YAP signal in EVT was significantly weakened after HCMV infection, and the nucleus location of the fluorescence signal was reduced (Fig. 6A, B). The decrease of YAP protein in the nucleus would downregulate the transcription of downstream genes, resulting in reduced proliferation of EVT and further invasion.

## Conclusions

In summary, HCMV reduces the proliferation and invasion of EVT by inhibiting Hippo signaling pathway, especially inhibiting the expression of YAP which serves as the downstream key effector in the pathway. Decreased invasion of EVT may affect the remodeling of the uterine spiral arteries and the blood perfusion of the placenta, further causing adverse pregnancy outcomes. However, the exact molecular mechanism needs to be further examined.

## Data Availability

All data generated or analysed during this study are included in this published article.

## Abbreviations

HCMV: Human cytomegalovirus
EVT: Extravillous cytotrophoblasts
HHV-5: Human herpes virus5
YAP: Yes-associated protein
CT: Cytotrophoblast
VT: Villous trophoblast
ST: Syncytiotrophoblast
